# Probiotics and Cancer: Mechanistic Insights and Organ-Specific Impact

**DOI:** 10.3390/biom15060879

**Published:** 2025-06-16

**Authors:** Md Faruque Ahmad, Fakhruddin Ali Ahmad, Abdulrahman A. Alsayegh, Md. Zeyaullah, Ahmad O. Babalghith, Hani Faidah, Faiyaz Ahmed, Anjum Khanam, Boshra Mozaffar, Nahla Kambal, Farkad Bantun

**Affiliations:** 1 Department of Clinical Nutrition, College of Nursing and Health Sciences, Jazan University, Jazan 45142, Saudi Arabia; aalsayegh@jazanu.edu.sa (A.A.A.); akhanam@jazanu.edu.sa (A.K.); bmozaffar@jazanu.edu.sa (B.M.); nhmohammed@jazanu.edu.sa (N.K.); 2 Department of Basic and Applied Science, School of Engineering and Science, G.D Goenka University, Gurugram 122103, Haryana, India; faahmad97@gmail.com; 3 Department of Basic Medical Science, College of Applied Medical Sciences, Khamis Mushayt Campus, King Khalid University (KKU), Abha 62561, Saudi Arabia; mdhafed@kku.edu.sa; 4 Department of Medical Genetics, Faculty of Medicine, Umm Al-Qura University, Makkah 24382, Saudi Arabia; aobabalghith@uqu.edu.sa; 5 Department of Microbiology and Parasitology, Faculty of Medicine, Umm Al-Qura University, Makkah 24382, Saudi Arabia; hsfaidah@uqu.edu.sa (H.F.); fmbantun@uqu.edu.sa (F.B.); 6 Department of Basic Health Sciences, College of Applied Medical Sciences, Qassim University, Buraydah 51452, Saudi Arabia; f.masfoor@qu.edu.sa

**Keywords:** probiotics in cancer, mechanisms, safety, chemotherapy, synergistic, antioxidant

## Abstract

Probiotics have been revealed in various studies to modulate the gut microbiome and have a substantial impact on cancers, comprising oesophageal, lung, liver, and colorectal cancer. These properties are endorsed by a diverse mechanism, including the modulation of the gut microbiome; preventing the metabolism of carcinogenic substances; exertion of anti-inflammatory action, immunopotentiator properties, and antioxidant activities; prevention of tumour growth; and decreasing the adverse effects of chemotherapy. There are promising perspectives regarding the new and developing field of probiotic research in relation to cancer treatment. This review demonstrates the recent findings of probiotics efficacy in cancer prevention and treatment and organ-specific impact along with protection from chemotherapy-induced side effects. The present evidence specifies that strategic probiotics application may be an effective complementary approach for the management of numerous kinds of cancer; still, additional studies and clinical trials are required to comprehend the relationships between cancer and probiotics.

## 1. Introduction

Cancer is a complicated group of disorders characterised by aberrant cell proliferation, which can have lethal effects on human health [1,2]. To decrease the worldwide cancer burden, ongoing research and improvements in prevention, initial detection, and treatment are required [3,4]. In most of the countries across the world, cancer is the leading cause of death [5]. By the year 2040, it is anticipated that cancer will be the cause of the deaths of around 16.3 million people [6]. Significantly, the level of social and economic development in a nation has a major impact on the incidence, prevalence, risk factors, and death rates associated with cancer [3,4,7]. Nearly 60% of the world’s population lives in Asia, where there are currently half of all cases and 58.3% of cancer-related deaths. Despite making up less than 10% of the world’s population, Europe is responsible for nearly one fifth of cancer cases and fatalities [4,8].

**Figure 1:**
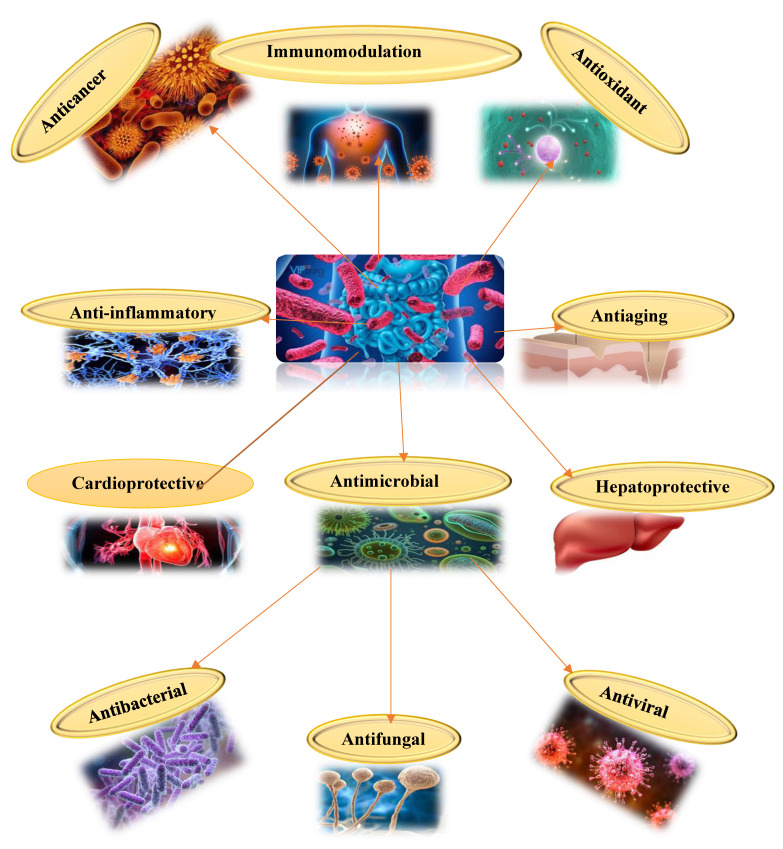
Exploring the various health promoting effects of probiotics, such as anticancer, antioxidant, anti-inflammatory, antiviral, antifungal, antibacterial, cardioprotective, hepatoprotective, health-promoting.

Probiotics are defined as live microorganisms that, when administered in adequate amounts, confer a health benefit on the host [9,10,11,12]. Probiotics have drawn a lot of curiosity regarding cancer in recent years, especially because of their potential to regulate the immune system and beneficial effects with anticancer activity [13]. The gut microbiome, a complex and diverse microbial community found in the human gut, plays an essential role in general health (Figure 1) [14]. Probiotics have the potential to aid in the maintenance of a balanced and healthy gut microbiome, which is important for many physiological functions and essential for preserving overall health, with an impact on the treatment of cancer [15,16,17]. Within the scientific field of probiotics, the genus *Lactobacillus* has been the subject of the most extensive research. Modern taxonomic revisions have divided *Lactobacillus* into 25 genera, improving our understanding of their functional diversity. The reclassification emphasises the need for precision in probiotic studies, particularly regarding anticancer implications [18,19]. This review examines the unique effects and mechanisms of probiotic strains in relation to various cancer types, along with safety precautions that may make them a viable adjunct therapy agent for cancer treatment and prevention.

## 2. Anticancer Mechanisms of Probiotics

Probiotics are receiving consideration due to their potential anticancer properties through multiple types of mechanisms of action. Below are some key ways that probiotics can help prevent and treat cancer [20,21,22,23].

### 2.1. Modulation of the Gastrointestinal Microbiota

One of the primary mechanisms by which probiotics can protect against the development of cancer is the modulation of the gastrointestinal microbiome [15,24]. The growth of pathogenic bacteria such as *Clostridium difficile*, *Escherichia coli* (*E.* *coli*), and *Fusobacterium* *nucleatum* can be selectively inhibited by probiotics, as they have been associated with an elevated risk of certain varieties of cancer [25,26,27]. Bacteriocins, organic acids, and hydrogen peroxide are some of the antimicrobial substances that are produced by probiotics in order to accomplish this goal. These substances have the potential to effectively restrict the growth of hazardous microbes [28,29]. Moreover, probiotics exhibit a significant role in preventing the growth of these pathogenic microbes by maintaining balance, differentiating the gut microbiome, and decreasing the probability of developing cancer. The metabolism of dietary ingredients, for instance, nitrosamines and bile acids, can change carcinogenic compounds [30]. Probiotics exhibit a significant role in decreasing carcinogenic substances in the body through regulating the enzymes involved in metabolic conversion [31,32]. Probiotics, which include species of *Lactobacillus* and *Bifidobacterium*, have the potential to prevent secondary bile acid production, which has been associated with developing colorectal cancer [23,33].

**Figure 2:**
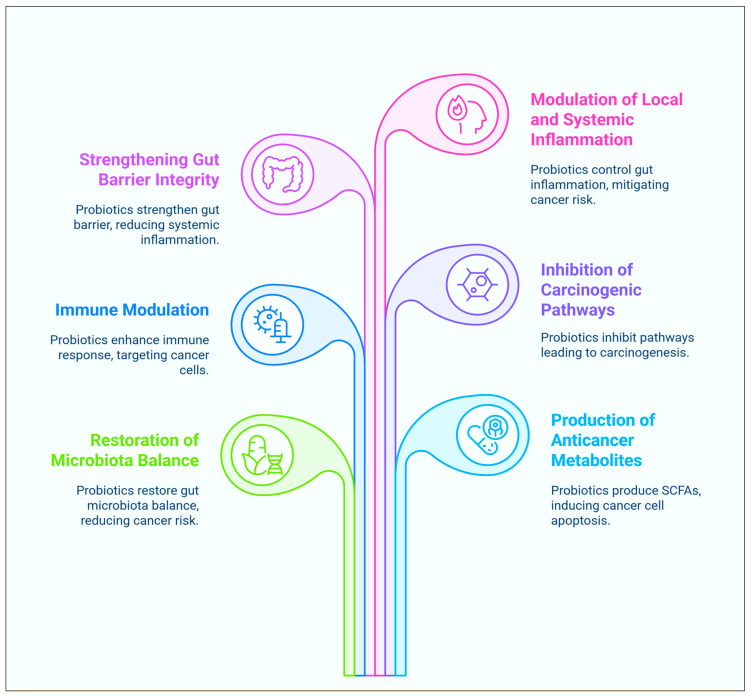
Probiotics anticancer mechanisms exhibited through modulation of gastrointestinal microbiota, such as strengthening gut barrier integrity, immune modulation of the gut microbiome, inhibition of carcinogenic pathways, production of anticancer metabolites, and restoration of microbiota balance.

The natural gut–liver axis balance can be recovered through probiotics application [34,35]. This is achieved through regulating the metabolism of bile acid, decreasing the levels of oxidative stress, and modifying the genes expression in liver cancer. Furthermore, probiotics can enhance the production of tight junction proteins such as occludin as well as claudins, improving the intestinal epithelial barrier. This barrier is crucial for controlling the passage of harmful substances, including carcinogens, from the intestinal lumen into the body [36,37]. They are a potential substance for maintaining the function of intestinal barrier system. Through improving intestinal barrier integrity, probiotics can reduce the host’s interaction with carcinogenic substances and prevent the systemic intake of these substances. Probiotics have the power to change the microbiome of the gut, which may help prevent and treat several types of cancer [38]. Various anticancer mechanisms through modulation of the gastrointestinal microbiota can be seen in Figure 2.

The composition of the baseline gut microbiota is a significant factor in influencing the efficacy of probiotics. Individual responses to probiotic treatment can be prominently influenced by alterations in the metabolic functions, microbiome diversity, and microbial taxa [39,40,41]. Better health outcomes are typically linked to greater microbial diversity. Probiotics may be more valuable for patients with a diversified microbiome since it can strengthened with supplementation of the preexisting microbial populations [42]. In one study, the baseline microbiome in postmenopausal women with obesity did not predict the therapeutic response to multispecies probiotics despite the fact that probiotics application did change the functional influence of the microbiota on metabolic markers [41]. The global microbiome variety and composition in healthy children remained relatively stable despite probiotic administration increasing the abundance of the supplemented strains. This suggests that baseline health status and microbiome stability may restrict the observable benefit of probiotics [42,43]. The composition of the microbiome can be further modulated by age, diet, and health state, which can affect the effectiveness of probiotics. Certain patient populations may benefit from probiotic therapies that are customised depending on these baseline traits [44]. While the concept of personalising probiotic treatments by considering patient-specific characteristics such as diets, genetics, and comorbidities is a matter of consideration; nevertheless, the present preclinical and clinical practice is not yet proficient at fully implementing this strategy. The most compelling evidence suggests tailoring treatment to the specific strain of probiotics and the various disease types under treatment. Even though there are studies that acknowledge factors such as obesity, diabetes, and dietary intake (for example, milk and yoghurt) as having an impact on the efficacy of probiotics, these factors have been considered in subgroup studies rather than as part of a personalised regimen [45,46,47].

### 2.2. Inhibition of the Metabolism of Carcinogenic Substances

Probiotics can establish an intestinal environment that is less favourable to the onset and spread of cancer by focusing on a variety of pathways [48]. One of the primary ways that probiotics can help prevent the development of cancer is by blocking the metabolism of carcinogens [49,50]. Some gut bacteria have enzymes that can break down environmental pollutants and food ingredients into compounds that cause cancer, like nitrosamines, polycyclic aromatic hydrocarbons, and heterocyclic amines [51,52]. The activity of these bacterial enzymes can be inhibited by probiotics such as *Lactobacillus* and *Bifidobacterium* species, which reduce the conversion of these compounds into their carcinogenic forms [53,54]. *Lactobacillus acidophilus* (*L. acidophilus*), for instance, has been demonstrated to impede the function of bacterial nitroreductases, which are responsible for transforming nitrosamines into more potent carcinogens [55,56,57]. Probiotics have the ability to affect the host enzyme activity involved in the metabolism of carcinogens. *Lactobacillus rhamnosus (L. rhamnosus)* and *Bifidobacterium* probiotic strains that have been shown to upregulate the expression of phase II detoxification enzymes, which are in charge of conjugating and excreting carcinogens [54]. These enzymes comprise glutathione S-transferases and UDP-glucuronosyltransferases. Through boosting the ability of the host to metabolise and remove carcinogens, probiotics have the potential to reduce the risk that carcinogens may either cause or accelerate the growth of cancer [58].

**Figure 3:**
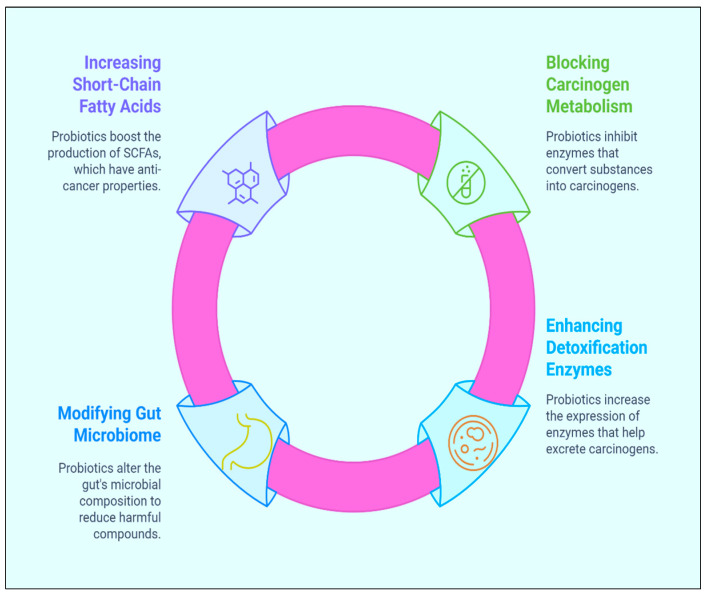
Inhibition of the metabolism of carcinogenic substances through preventing carcinogenic metabolism, modifying the gut microbiome, improving detoxification enzymes, and increasing the short-chain fatty acids.

Heterocyclic amines and secondary bile acids are two examples of foods that can be transformed into carcinogenic metabolites. These metabolites are produced by particular bacteria that live in the gut. Probiotics have the ability to change the composition of the gut microbiome as well as its metabolism, which in turn reduces the production of compounds that are poisonous to the body [59,60]. *Lactobacillus* and * Bifidobacterium* species have been demonstrated to impede the process of the 7α-dehydroxylation of primary bile acids. This is one of the most important steps in the production of secondary bile acids, which have been associated with an increased probability of developing colorectal cancer [61,62]. Furthermore, it has been demonstrated that short-chain fatty acids (SCFAs), such as butyrate, acetate, and propionate, exert activities that inhibit the development of cancer. The production of these SCFAs can be increased by the use of probiotics [63,64]. These short-chain fatty acids have the potential to alter epigenetic pathways that regulate the expression of tumour suppressor genes and oncogenes. Furthermore, they have the ability to inhibit the proliferation of cancer cells and promote cell death through apoptosis [65,66]. Overall, one of the most significant ways that probiotics can assist in the prevention of cancer is by preventing the metabolism of potentially cancer-causing substances (Figure 3) [67,68].

### 2.3. Limiting the Growth and Proliferation of Tumours

**Figure 4:**
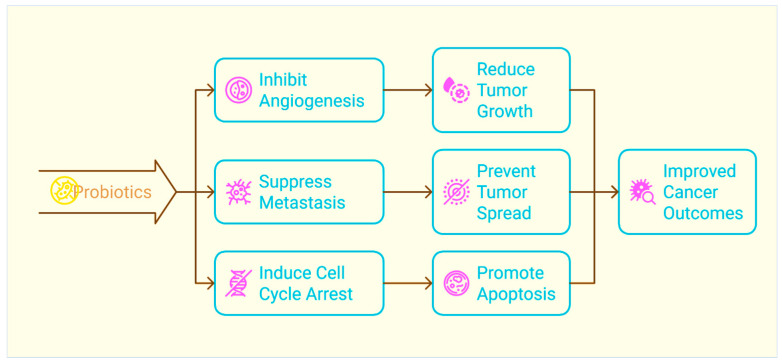
Restricting the growth and proliferation of tumours via suppressing metastasis, inducing cell cycle arrest, and inhibiting angiogenesis.

Angiogenesis, the formation of new blood vessels, is essential for the growth and spread of solid tumours. Probiotics have been shown to inhibit the production of pro-angiogenic factors essential for the formation of new blood vessels [69,70]. Matrix metalloproteinases (MMPs) and vascular endothelial growth factor (VEGF) are two examples of these factors. Probiotics have the potential to prevent the malignancy from acquiring the oxygen and nutrients necessary for growth and dissemination by inhibiting the process of angiogenesis [71,72]. Among the molecules that are involved in the process of metastatic spread, probiotics have the ability to inhibit the expression of specific molecules. These molecules include adhesion molecules, which are responsible for mediating the attachment of tumour cells to the vascular endothelium, and matrix metalloproteinases (MMPs), which contribute to the breakdown of the extracellular matrix. Through their ability to inhibit the mechanisms that contribute to the development of metastasis, probiotics have the potential to reduce the ability of tumour cells to infiltrate, migrate, and establish secondary tumours in other locations [21,73,74]. One of the most significant stages of metastasis is the epithelial–mesenchymal transition (EMT), which occurs in the early stages of the disease. During this phase, epithelial cells lose their polarity and their ability to adhere to other cells, and they evolve a phenotype that is more invasive and more likely to migrate [75]. Probiotics may hinder the EMT progression by preventing the expression of transcription factors [76,77]. As a result of their ability to suppress the EMT, probiotics have the potential to impede the ability of tumour cells to spread from the primary site, infiltrate through the extracellular matrix, and establish secondary tumours in different areas [78,79]. It has been demonstrated that certain strains of probiotics, such as *L. acidophilus*, have the ability to directly suppress the growth of cancer cells by triggering cell cycle arrest and apoptosis [80,81]. *L. acidophilus* may inhibit cancer cell proliferation by cell cycle arrest and apoptosis, involving several key genes. The p21 (CDKN1A) cyclin-dependent kinase inhibitor is important for inducing cell cycle arrest by impeding cyclin–CDK complexes, particularly during the G1 phase of the cell cycle [82]. Like p21, p27 is a cyclin-dependent kinase inhibitor that controls the cell cycle by preventing progression from the G1 to the S phase. It also participates in facilitating cell cycle arrest. Bax, a pro-apoptotic gene, is commonly improved during apoptosis, facilitating cell death in cancer cells [82]. This impact is produced by the formation of SCFAs such as propionate and butyrate, which have the ability to influence the expression of genes associated with regulation of the cell cycle and apoptosis (Figure 4) [83,84].

### 2.4. Anti-Inflammatory and Immunopotentiators Efficacy

**Figure 5:**
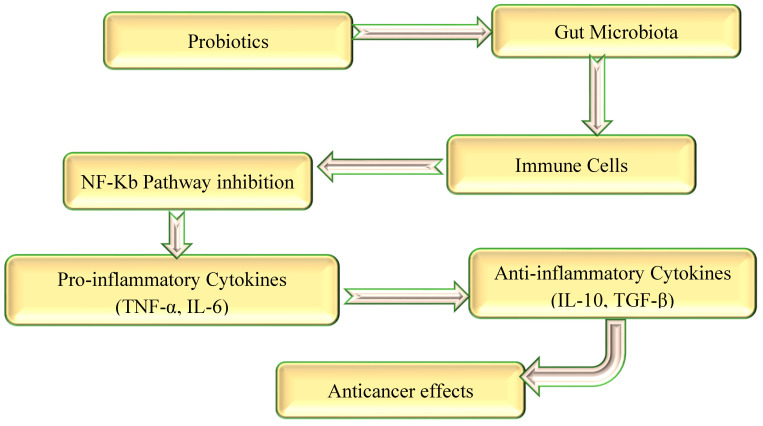
Diagram illustrating precisely how probiotics interact with the immune system to provide anti-inflammatory and anticancer benefits. First, probiotics affect the gut microbiota, which is essential for regulating the immune system. Through the inhibition of the NFKB pathway, this interaction affects different immune cells and regulates inflammatory pathways. The inhibition of this pathway consequences lead to release of pro-inflammatory cytokines, which then triggers the anti-inflammatory cytokines. These anti-inflammatory signals help to control the inflammation, ultimately forming a less favourable atmosphere for cancer development and endorsing an anticancerous effect.

Probiotics and their potential effects on immunity in relation to cancer are currently the subject of ongoing research. Probiotics may strengthen the immune system, according to the results of certain studies, which could have a beneficial impact on the treatment process for cancer as well as the progression of the disease. Through the enhancement of the immune system of the host, probiotics have the ability to inhibit the growth and spread of tumour cells, hence facilitating the identification and elimination of tumour cells [85,86]. However, probiotics have the ability to change the microenvironment of tumours by reducing immune-suppressive cells, such as myeloid-derived suppressor cells and regulatory T cells, which are known to be able to hamper the immune response against tumours [87]. Probiotics and their metabolites significantly modulate the tumour microenvironment, which exhibits a specific impact on immune cells in situ. By encouraging the activities of numerous immune cells, like natural killer cells and T cells, they enhance the immune response to tumours. They also alter the ratio of cytokines (pro-inflammatory to anti-inflammatory), which is important for immunological homeostasis [88]. The immunological landscape of the tumour can be affected by metabolites, for instance, SCFAs, which can prevent pro-tumorigenic immune cells and increase the proliferation of regulatory T cells (Tregs) A. Furthermore, probiotics can change the composition of the extracellular matrix by interactions with stromal cells, including fibroblasts and endothelial cells, creating an environment that is more favourable to antitumour immunity [88]. Moreover, stromal cells metabolic state may be affected by probiotic metabolites, which may result in modifications that support tumour destruction [88]. Furthermore, it has been determined that metabolites such as SCFAs and microbial tryptophan catabolites exhibit an essential role in regulating the TME, enhancing antitumour immune responses, and affecting immune cell action [89]. Through a variety of signalling pathways, numerous probiotics may potentially directly interact with tumour cells, triggering apoptosis or preventing proliferation [88,89]. Probiotics enhance the production of cytokines, such as interleukin-2 (IL-2), interleukin-12 (IL-12), and interferon-gamma (IFN-γ), that can provide support for the antitumour actions of immune cells [90]. When probiotics are consumed, there is an increase in the expression of molecules belonging to the major histocompatibility complex (MHC) class I and class II. This may result in an improvement in the immune system’s abilities to recognise and eradicate cancer cells [91]. In addition, intestinal epithelial cells, which act as a barrier against pathogens and contribute to the maintenance of immunological homeostasis, have the potential to function more effectively when probiotics are integrated into their diet [92]. The production of inflammatory mediators, such as prostaglandins, leukotrienes, and cytokines, is usually high in cancer patients [93]. Probiotics have the potential to regulate the production of these mediators, which are frequently elevated in cancer patients. They can prevent essential inflammatory pathways that are implicated in the beginning and progression of cancer, including as the NF-κB and MAPK signalling cascades [94,95]. They have the ability to block essential inflammatory pathways from being activated. Furthermore, it is worth noting that probiotics possess the capability to enhance the expression of anti-inflammatory molecules such as TGF-β and IL-10, which might be of great assistance in combating the pro-inflammatory state that is associated with cancer [96,97]. There is evidence that probiotics have the ability to inhibit the activation of inflammatory signalling pathways, which are critical for the growth of tumours, the formation of new blood vessels, and the spread of metastases. The COX-2/PGE2 pathway is recognised as one of these pathways [81]. The gut microbiome’s ability to be modulated by probiotics can also affect the synthesis of bacterial metabolites, like SCFAs, which have anti-inflammatory and anticancer properties. Probiotics can help stop the development and spread of several cancers, such as colorectal, lung, liver, breast, and prostate cancer, by lowering chronic inflammation (Figure 5) [98,99].

### 2.5. Antioxidant Properties

**Figure 6:**
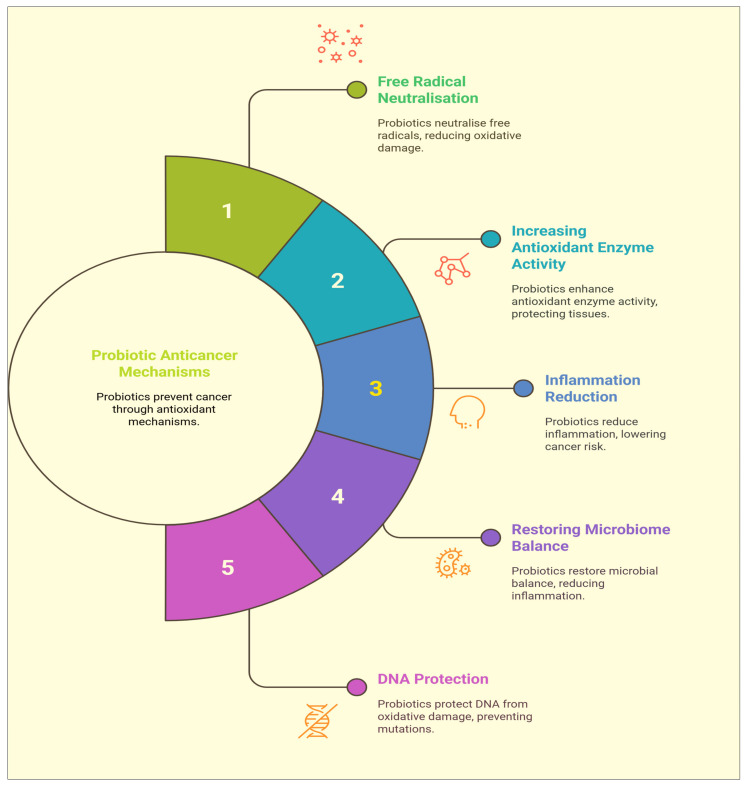
Probiotics exhibit anticancer properties in different ways, such as neutralising free radicals, increasing antioxidant enzymes, reducing inflammation, restoring microbial balance, protecting DNA, lowering oxidative stress, and inhibiting carcinogens.

Reactive oxygen species and free radicals can harm lipids, proteins, and DNA. This can result in genetic mutations, genomic instability, and cellular dysfunction, all of which can fuel the development and spread of cancer [100,101]. The antioxidants found in probiotics may protect DNA from oxidative damage, lowering the possibility of mutations and genomic instability that can lead to the emergence of cancer. Probiotics antioxidant properties are additionally useful in the suppression of metastasis and angiogenesis [54,102]. This potential antioxidant ability has been demonstrated across different probiotic strains, especially those belonging to the *Lactobacillus* and *Bifidobacterium* genera. Reactive oxygen species (ROS), free radicals, and other oxidizing agents can be directly scavenged and neutralized by probiotics, stopping them from damaging cells [54,103]. Nuclear factor erythroid 2-related factor 2 (Nrf2) is one example of a transcription factor and signalling pathway whose activity is influenced by probiotics and regulates the expression of antioxidant genes [104,105]. Pro-oxidant metal ions, like iron and copper, can be chelated by some probiotics to prevent them from catalysing reactions, which would result in the production of free radicals. Probiotic antioxidants can hinder the development and spread of several cancers, such as colorectal, breast, and prostate cancer, by lowering oxidative stress [106,107,108,109]. Probiotics can further strengthen the body’s antioxidant defences by increasing the activity of endogenous antioxidant enzymes like catalase, superoxide dismutase, and glutathione peroxidase [110]. Several studies have revealed that probiotic supplements may help cancer patients experience better treatment outcomes by lowering oxidative stress and increasing antioxidant status [106,110] (Figure 6).

Antioxidants from probiotics may decrease the side effects and increase patient tolerance of cancer treatments like radiation and chemotherapy [102,111]. They reduce oxidative stress and inflammation caused by chemotherapy therapies, and they may work in conjunction therapy along with chemotherapy. Since probiotic-derived antioxidants target multiple pathways involved in cancer progression, they may also help prevent the development of resistance to standard cancer treatments [112,113].

## 3. The Role of Probiotics in Cancer Management

Probiotics’ role in cancer management and prevention is still growing despite the abundance of ongoing research in this area. Probiotics are being investigated in this context because there is mounting evidence to support the critical role the gut microbiota plays in many facets of human health. Table 1 presents a more thorough summary of how probiotics may be used in relation to various cancer types.

### 3.1. Oral and Esophageal Cancer

Probiotics have emerged as a significant study focus in oral cancer, mainly for their ability to influence the oral microbiome and improve therapy results. Probiotics like *Lactobacillus* and *Bifidobacterium* may balance the oral microbiome, thereby reducing oral cancer risk by suppressing harmful pathogens and increasing favourable bacteria [114]. Probiotics, in combination with prebiotics, have demonstrated potential in the management of oral potentially malignant disorders (OPMDs) [115]. Probiotics have been thoroughly investigated for their efficacy in preventing and alleviating the severity of oral mucositis (OM), a prevalent and severe consequence of cancer treatments such as chemotherapy and radiotherapy. Numerous studies indicate that probiotic supplementation markedly reduces the frequency and severity of oral mucositis (OM), especially severe oral mucositis (SOM), in cancer patients [115,116,117]. Probiotics effectively diminish the occurrence and severity of oral mucositis in cancer patients, presumably via immunomodulatory and microbiota-regulating mechanisms [118]. The evidence supporting the direct anticancer benefits of probiotics on oral carcinogenesis is scarce and predominantly derived from in vitro and animal studies. Specific probiotic strains, including *Lactobacillus salivarius* Ren and *Lactobacillus plantarum*, have shown efficacy in inhibiting the proliferation of oral cancer cells and diminishing the risk of oral cancer development in preclinical settings. Nonetheless, there is inadequate clinical evidence that probiotics can directly prevent or treat oral cancer in human [118,119]. Although preclinical research has demonstrated their anticancer properties, more clinical studies are needed to explore probiotics in the management of oral cancer.

Oesophageal cancer is a perilous form of malignancy that originates in the oesophagus. It has an unfavourable prognosis and can be difficult to manage therapeutically. Specific probiotic strains may confer advantages for individuals with oesophageal cancer, as indicated by specific studies [120]. According to a systematic review, patients with digestive cancer and control subjects had different oral microbiota compositions [121]. Accordingly, it has been proposed that the oral microbiome could affect the risk of developing oesophageal cancer [120,122,123]. Peters et al. (2017) identified a correlation between *Tannerella forsythia* (*T. forsythia*) and an elevated risk of oesophageal adenocarcinoma (EAC), as well as a link between *Porphyromonas gingivalis* and an increased risk of oesophageal squamous cell carcinoma (ESCC). Additionally, it was noted that a decreased risk of EAC may be linked to decreased levels of *Neisseria* and *Streptococcus pneumonia* [122]. In a recent study, Kawasaki et al. (2021) conducted an investigation into the relationship between oesophageal cancer and the oral microbial culture [124]. In this study, 61 patients with oesophageal cancer and 62 matched individuals (without cancer) participated. In order to investigate the oral microbiome, saliva samples that were not stimulated and subgingival plaque samples were obtained. The results of the study suggest that there may be a correlation between specific bacteria and an elevated risk of oesophageal cancer. These bacteria are *Aggregatibacter actinomycetemcomitans* from unstimulated saliva and *T. forsythia* and *Streptococcus anginosus* from dental plaque [124].

A study conducted by Liu et al. (2020) investigated the link between the oral microbiome and a possibility of developing malignant oesophageal lesions [120]. 16S rDNA gene sequencing was applied to examine the microbiome. The findings suggest that the oral microbiome significantly influences oesophageal cancer and may serve as a valuable indicator for early diagnosis. A parallel study was conducted on 39 oesophageal cancer patients and 51 volunteers serving as the control group in a comparable study in a comparable part of the world [125]. The 16S rDNA gene sequencing method was used to analyse the oral microbiome. The study also identified variations in the oral microbiome between oesophageal cancer patients and healthy individuals. *Remarkably*, *Selenomonadales*, *Negativicutes*, *Prevotellaceae*, and *Prevotella* were discovered in higher levels in oesophageal cancer patients than in healthy individuals, while *Betaproteobacteria*, *Proteobacteria*, and *Neisseria* had a lower percentage [125].

Probiotics can be given as an additional therapy for oesophageal cancer in order to enhance the immune system’s performance and nutritional status, among other benefits [126]. In a double-blind, randomized, placebo-controlled study involving cancer patients (including those with oesophageal cancer), the effect of probiotics on their nutritional intake was examined [127]. *Lactobacillus plantarum* (*L. plantarum*) 299v, a probiotic strain, was administered during this research. Albumin levels were found to be significantly higher in the probiotic-receiving group. Albumin levels, along with prealbumin, total protein levels, and overall lymphocytes count, are among the laboratory variables used to assess nutritional status [127]. According to a study by Hashemi-Khah et al. (2022), a different probiotic called *L. rhamnosus* (PTCC 1637) could additionally be beneficial in the event of oesophageal cancer [128], although *Lactobacillus* and *Bifidobacterium* genera are the most widely used probiotics.

**Table 1:** Different microbial strains with anticancer properties.

Types of Cancer	Probiotic Strains	Mechanisms	Remarks	References
Colon cancer	*Lactobacillus plantarum* YYC-3	The mechanism involves the modification of the immune system, resulting in the downregulation of inflammatory cytokines interleukin (IL)-6, IL-17F, and IL-22 as well as a decrease in the infiltration of inflammatory cells.	Suppressed colon cancer cell lines and averted tumour development by altering immune response and gut microbiota.	[129]
Colorectal cancer	*L. rhamnosus*	Improves the functioning of the gut barrier, controls the immune system, and stops the growth of tumours.	Studies on animals revealed a lower incidence of tumours. May boost digestive system in general.	[130,131,132]
	*B. longum* and *L. acidophilus*	Generate butyrate and other SCFAs, which have anti-inflammatory properties; strengthen the body’s defences against cancer.	SCFAs have the ability to trigger apoptosis and stop the growth of cancer cells.	
Liver cancer	*L. rhamnosus*	Reduces liver inflammation and fibrosis by altering the gut microbiota.	There is little proof; more research is needed to confirm the effects.	[133,134]
Gastric cancer	*L. acidophilus*	Generates lactic acid, which inhibits harmful microorganisms and lowers the pH of the stomach.	May contribute to gut health improvement and a reduction in stomach inflammation.	[135]
	*L. casei*	Boosts the immune system and may lessen stomach mucosal irritation.	Few studies have been conducted; additional study is required to confirm effects on stomach cancer.	[136].
	*L. plantarum*	Improves the immunological response and changes the composition of the gut microbiome.	Potential advantages in lowering inflammation and enhancing gut health were suggested by research.	[137]
Breast cancer	*L. acidophilus*	Alters the metabolism of oestrogen, which may lower oestrogen levels and strengthens the body’s defences against cancerous cells.	There may be a connection between breast cancer risk and gut bacteria, according to some research.	[138]
	*L. casei*	Improves the immune system and, by modifying cytokines, may slow the growth of tumours.	Few studies have been conducted, research is still ongoing, while primary outcomes are promising, additional extensive clinical trials are required to establish certain effects and mechanisms in breast cancer.	[139]
Bladder cancer	*L. acidophilus*	Creates lactic acid, which can support the preservation of a healthy environment in the urinary system.	Assisted in reducing the incidence of urinary tract infections, which may minimize the risk of bladder cancer.	[140]
	*Bifidobacterium longum*	Reveals anti-angiogenesis and antiproliferation of bladder and stomach cancer.	Few studies have been conducted; additional study is required to confirm effects on stomach cancer.	[141]
Oesophageal cancer	*L. rhamnosus*	Strengthens the immune system and may aid in lessening oesophageal irritation.	Enhancing gut health may have an indirect impact on oesophageal health, according to some research.	[128]
Oral cancer	*Lactobacillus salivarius* (*L. salivarius*)	Downregulates cyclooxygenase 2 (COX-2) expression, prohibits DNA from oxidative injury, and suppresses tumorigenesis.	Antiproliferative and apoptotic action of *L. salivarius* reported in oral cancer cell.	[142,143]
Cancer therapy-induced oral mucositis	*Lactobacillus* CD2, *Bifidobacterium*, and *Lactobacillus* species	Improve oral tissue repair, decrease inflammation, and strengthen mucosal immunity.	The application of probiotics in cancer therapy patients resulted in a reduction in both the incidence and severity of oral mucositis.	[144,145]
Prostate cancer	*Lactobacillus acidophilus*	Probiotics, including *Lactobacillus acidophilus*, contribute to regulating rectal volume fluctuations during radiation therapy for prostate cancer by altering gut microbiota, diminishing inflammation, and improving mucosal barrier integrity.	This resulted in reduced rectal volume fluctuations and enhanced tolerance to radiation therapy, ultimately stabilising prostate placement during treatment.	[146]
Breast cancer	*Lactobacillus casei* strains	Prevent the growth of breast tumours, reduce metastases, and prolong survival.	Minimized immunosuppression in metastatic regions while preserving a balanced inflammatory response.	[147,148]
Pancreatic cancer	*Lactobacillus casei* and *Aspergillus oryzae*	Promote apoptosis and dysregulation of the cell cycle and p38 MAPK pathway activation.	The probiotic exhibited its ability to prevent the proliferation of pancreatic cancer cells.	[149,150]
Various different cancers	*Lactobacillus rhamnosus*, *Bifidobacterium longum*, and *Lactobacillus casei*	These probiotics reduce inflammation and encourage cancer cell death by enhancing immune responses, altering the gut microbiota, and inactivating carcinogenic substances.	These probiotics modulated the microbiota in the gut and the immune response, making them effective adjuvants in the treatment and prevention of cancer.	[151,152]

### 3.2. Colon Cancer

Probiotics have been extensively studied for their potential role in the treatment and prevention of colon cancer and have received important focus in recent years [50,153]. According to numerous studies, taking probiotics on a regular basis may enhance the intestinal microbiota’s quantitative and qualitative profile, which would lessen the development of carcinogenic compounds during intestinal dysbiosis and the trigger for chronic inflammation [154,155,156]. Patients with colorectal cancer (CRC) undergoing a 16-day colorectomy showed an increase in microbial numbers and diversity when they regularly consumed high dosages of *L. plantarum*, *L. acidophilus*, and *B. longum* [20,156]. The intestinal microbiota composition of the patients in this study was similar to that of the healthy subjects. Certain intestinal enzymes, including β-glucosidase, azoredutase, 7-α-dehydroxylase, and β-glucuronidase, have cytotoxic and genotoxic properties, which can contribute to the emergence of colon cancer by converting aromatic hydrocarbons and amines into active carcinogens through the synthesis of phenols, ammonia, aglycones, and N-nitroso compounds [157]. It has been reported in study that consuming specific probiotic bacterial strains can lower these enzymes’ activity and avert colon cancer. Some probiotics can also affect the immune response by stimulating phagocytes, which helps to maintain an immune-vigilant state that can eradicate cancer cells at an early stage in their growth. It is important to remember that the immunomodulatory qualities depend on the strain; the immune system can also be significantly impacted by the bacteria’s ability to survive and persist in the gastrointestinal tract as well as their posology [20,158,159].

**Figure 7:**
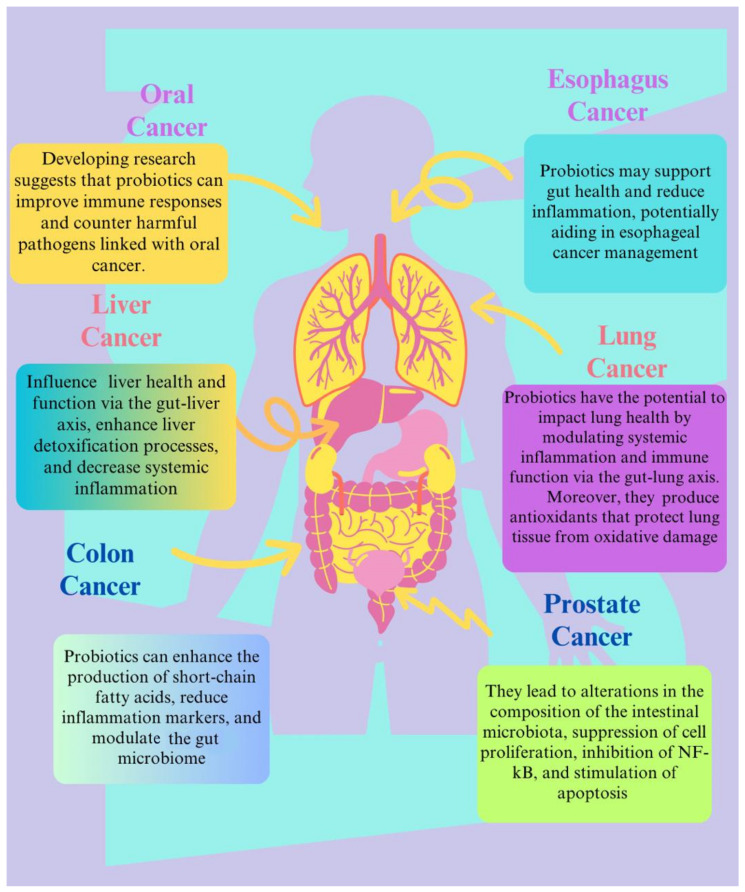
Probiotics’ potential to influence systemic health may contribute to the treatment of numerous cancers, such as oral, oesophageal, liver, colon, lung, and prostate, across different organs and through different modes of action.

Consequently, not all probiotics have the ability to alter the immune system and stop CC from occurring [153]. However, frequent use of probiotics has been shown to decrease the permeability of the gut by altering the flow of cell junction proteins and lowering the absorption of potentially cancer-causing substances [160,161]. Probiotic treatment (*L. acidophilus*, *B. longum*, and *L. plantarum*) enhanced the prognosis and increased the amount and distribution of cell junction proteins in the colonic epithelium of CRC patients [156]. Moreover, probiotics can help to repair the intestinal barrier, reduce inflammation in the gastrointestinal tract, and maintain gut homeostasis; in particular, *Clostridium butyricum* (*C. butyricum*) has been shown to regulate gut homeostasis, reduce inflammation, and reduce the frequency of diarrhoea in digestive diseases like inflammatory bowel disease (IBD), and it has been used to treat gastrointestinal diseases [162,163,164]. Rafter et al. (2007) found that oral therapy with a combination of *L. rhamnosus* and *B. breve* reduced multiple cancer biomarkers as well as genotoxic exposure (IL-2 and INF-gamma) [131]. Kotzampassi et al. (2015) found that using a probiotic formulation significantly reduced major complications following surgery in individuals with colon and rectal cancer [165]. Probiotics’ anticancer properties against various organs are depicted in Figure 7.

### 3.3. Hepatocellular Carcinoma

Liver cancer, or hepatocellular carcinoma (HCC), is among the most prevalent and deadly cancers in the world, with an increasing incidence in numerous nations [166]. Probiotic supplements, such as those containing *Lactobacillus* and *Bifidobacterium* strains, may contribute to restore a balanced gut microbiome. A healthy gut microbiome is essential for regulating immune function, inflammation, and metabolic processes, all of which can influence the occurrence and progression of HCC [167]. HCC patients’ treatment options are determined by the stage of the disease, liver function, and cost. Although patients with HCC have a higher survival rate, recurrence remains a major concern for them [168]. Many liver diseases cause changes in the composition of the gut microbial community; therefore, reorganizing the microbiota in the gut may offer a new avenue for treatment. Probiotics have been shown to have positive effects on liver and gastrointestinal disorders [9,169]. Probiotics have been shown to reduce the risk of HCC in a number of studies [170,171,172]. Li et al. (2016) stated that probiotics prevent the progression of HCC in mice. Feeding the probiotic mixture Prohep (which contains *E. coli* Nissle 1917, *L. rhamnosus* GG, and heat-inactivated VSL#3) to tumour-injected mice may alter the gut microbiota composition while decreasing the size of liver tumours and also down-regulating angiogenic factors [173].

Exposure to mycotoxins may cause damage to the intestinal epithelia, but probiotic bacteria may mitigate this effect. Trans-epithelial electrical resistance (TEER) was significantly reduced when aflatoxin B1 (AFB1) was incubated with CYP3A4-induced Caco-2 monolayers [174]. The aflatoxin-induced decrease in TEER was lessened when probiotic bacteria were present, suggesting that probiotic bacteria may lessen cytotoxicity induced by aflatoxin. Probiotic dietary supplements were also demonstrated to successfully lower urinary elimination of aflatoxin-DNA adduct (AFB1-N7-guanine), a reliable biomarker for the risk of liver cancer, in Chinese subjects exposed to AFB1 [174]. These findings imply that taking probiotic supplements lowers the risk of HCC by reducing the bioavailability of the carcinogen AFB1.

**Figure 8:**
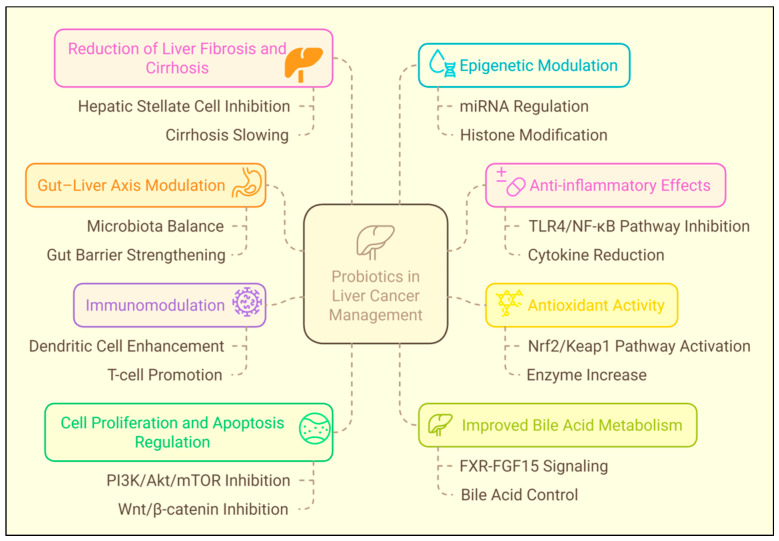
Through controlling inflammation, immunological responses, and metabolic processes, the gut–liver axis plays a critical role in preventing liver cancer, illustrating the need of preserving a healthy gut microbiome. Furthermore, it has been demonstrated that probiotics affect a number of signalling pathways that may be involved in the management of liver cancer. These pathways are essential for controlling apoptosis and inflammation.

Incorporating probiotics into a comprehensive treatment strategy for liver cancer may allow medical professionals to improve patient outcomes, enhance quality of life, and uncover novel approaches to using the gut–liver axis in the clinical management of cancer [169]. On the other hand, we need to conduct additional study in order to identify the probiotic strains, dosages, and delivery systems that are most effective for patients with liver cancer. It is possible that in order to maximise the potential benefits of probiotics in the management of liver cancer, a customised strategy that is based on the specific characteristics of the patient is required. The mechanisms of probiotics in liver cancer are illustrated in Figure 8.

### 3.4. Lung Cancer

Worldwide, lung cancer remains a significant reason of cancer-related deaths [175]. An abundance of evidence has demonstrated the critical roles that probiotics play in various forms of tumour prevention and treatment [85]. Although there is currently limited proof supporting the role of probiotics in lung cancer prevention, a few studies have indicated a promising role for probiotics [176,177,178]. One study suggested that well-balanced intestinal microflora may play a protective role in the management of lung cancer [179]. Probiotics can contribute to the preservation of a healthy and balanced gut microbiome, which in turn can have a beneficial impact on the lung microbiome through the gut–lung axis [180]. A healthy gut–lung axis can lower inflammation and enhance immunological response, which lowers the risk of developing lung cancer [180,181]. Additionally, probiotics can stimulate the production of antimicrobial peptides and other immune-boosting substances as well as improve the body’s capacity to identify and eradicate lung cancer cells by enhancing immune cell function and activity like T cells and natural killer cells [182]. Probiotics with antioxidant properties can help to reduce the risk of lung cancer, considering that oxidative stress is a contributing factor in the disease’s development [183].

Probiotics were utilized as tumour models in one in vivo study using Lewis lung cancer (LLC)-bearing mice (C57BL/6J). A cisplatin group, A cisplatin/ABX group (an antibiotic combination of ampicillin, neomycin, and vancomycin that disrupts intestinal microflora homeostasis), and a probiotic group cisplatin/*L. acidophilus* were the three treatment groups to which the lung cancer cells were assigned. Positive outcomes included smaller tumours and higher survival rates. The mice in the cisplatin and cisplatin/ABX groups had reduced survival rates, while the mice in the cisplatin/*L. acidophilus* group had greater survival rates [184]. In another study, the efficacy of an antitumour vaccine supplemented with probiotics or their products was assessed for solid sarcoma 37 (S37) and metastatic Lewis lung carcinoma. They used a probiotic mixture made up of *Enterococcus faecium* K-50 and *Saccharomyces cerevisiae* as well as their metabolic products. The findings showed that the simultaneous administration of the antitumour vaccine with probiotics and/or prebiotics produced a synergistic impact in therapy for S37-bearing mice, and in 3LL-bearing animals, the collective application prohibited metastasis by 2 to 2.5 times compared to animals treated with vaccine alone [185].

**Figure 9:**
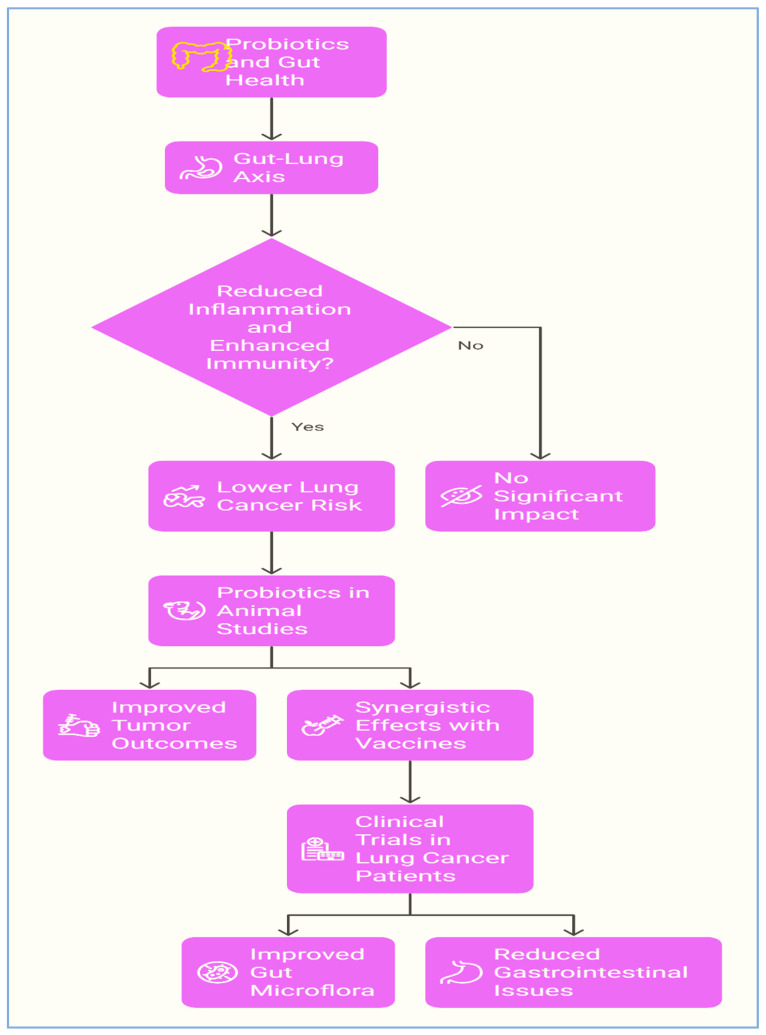
Significant roles of probiotics in management of lung cancer. Probiotics may improve immune modulation, decrease inflammation, support the gut–lung axis, and improve the gut microbiota, helping in the metabolism of carcinogens and ultimately contributing to improved survival rates, quality of life, and treatment efficiency for lung cancer patients.

A clinical trial on 30 lung cancer participants was conducted to investigate the possibility of improving gut bacteria in patients receiving chemotherapy along with probiotic strains. One group (*n* = 21) that received a combination treatment of a probiotic strain based on *Bacillus subtilis* and chemotherapy showed an improvement in intestinal microflora and a lower rate of intestinal dyspepsia. Patients in the control group (*n* = 9) who only received chemotherapy experienced constipation; a decrease in *Bifidobacterium*, *Lactobacillus*, and *Bacteroides*; and elevated levels of various strains of pathogenic bacteria [186]. Using this combination (probiotics and chemotherapy) in lung cancer patients can reduce the frequency of gastrointestinal complaints and prevent gut microflora degeneration (Figure 9).

### 3.5. Breast Cancer

The application of probiotics as an adjunctive strategy in the management of breast cancer is now being explored. This research focusses on the possible functions that probiotics may play in preventing, treating, and lessening side effects associated with treatment [187,188]. A recent study and clinical trials have explored an extensive range of probiotic strains and combinations, analysing the impact of these probiotics on cancer and the quality of life of patients with breast cancer. Several studies have proven that probiotics have the potential to influence the immune system, which may result in increased antitumour effects and decreased inflammation. They may also have an effect on the microbiota of the breast and the gut, both of which have been associated with the development and progression of cancer [188,189,190]. Probiotics exhibit a potential role in apoptosis of breast cancer, restricting its proliferation and diminishing tumour growth and metastasis [190,191]. A combination of probiotics, including *Lactobacillus*, *Enterococcus*, and *Bifidobacterium*, helps to combat dyslipidaemia, metabolic disorders, and obesity, which have been linked with breast cancer and its therapy. Furthermore, the composition of the gut microbiota can be altered, which has been associated with higher inflammatory profiles and decreased levels of pro-inflammatory markers such as TNF-α [189,191,192]. Probiotics have been shown to have an effect on plasma metabolites, such as p-Mentha-1,8-dien-7-ol, which have been linked to reduced toxicity from chemotherapy as well as improved neurological and metabolic outcomes [191,192]. According to certain human studies, using fermented dairy products and particular probiotics like *Lactobacillus casei* Shirota consistently may decrease the risk of breast cancer [187]. Additionally, better results against chemotherapy-induced oral mucositis and cognitive impairment have been reported as well as an overall improvement in the quality of life during treatment [189,191,192]. Probiotics have the potential to supplement conventional treatments by enhancing immunological and metabolic health [188,189,193]. Supplementation works better than probiotics alone, even though probiotics are often taken with prebiotics during chemotherapy cycles [193]. Probiotics have the potential to prevent and treat breast cancer, especially by lowering adverse effects from treatment and enhancing immunological and metabolic function. Large-scale, effectively planned clinical trials are required to demonstrate efficacy and safety in humans, as most of the data for direct anticancer effects come from animal studies [187,188].

### 3.6. Prostate Cancer

Probiotics have garnered interest as potential agents to control the risk of prostate cancer and progression, mostly as a result of their impact on the gut microbiome [194]. Various research includes preclinical, cellular, and animal studies, and initial clinical study confirmed that probiotics produce significant effects in the management of prostate cancer [195]. The research evaluated the antiproliferative and apoptotic efficacy of probiotic whey dairy beverages on human prostate cancer cell lines. Five whey beverages were evaluated, comprising traditional whey drinks and those supplemented with probiotic microorganisms. Probiotic whey beverages with strains such as *L. acidophilus, Bifidobacterium animalis,* and *L. casei* declined the viability and activated apoptosis in prostate cancer cell lines (PC-3 and DU-145) [196]. An in vitro investigation indicated that a probiotic-derived substance, specifically 1,4-dihydroxy-2-naphthoic acid (DHNA), exhibited preferential toxicity towards metastatic prostate cancer cells, diminished colony formation, and impeded cell migration and proliferation [197]. Additionally, the trial investigated the efficacy of an oral probiotic supplement in enhancing intestinal health and addressing benign prostatic hyperplasia (BPH) [146]. *Bifidobacterium longum* and *Bifidobacterium psychaerophilum* demonstrated a correlation within the gut microbiota. Probiotics regulated essential mediators of BPH and diminished inflammation and oxidative stress in simulated gut–prostate axis models. In addition, probiotics exhibited a potential role as an adjunct to radiation therapy. The use of *L. acidophilus* as a supplement reduced variations in rectal volume during radiation therapy for prostate cancer, which may have contributed to an improvement in treatment efficacy [146]. Despite the fact that a trial utilising probiotics based on *Pediococcus* did not show any significant impact on rectal volume or quality of life, the intervention was found to be safe and viable [146].

Probiotics exhibit a potential role in altering the microbiota composition of gastrointestinal tract, which is linked with the growth of prostate cancer through influencing systemic hormone levels, immune responses, and inflammation. Prostate cancer pathogenesis is influenced by particular bacteria such as *Prausnitzii*, *Faecalibacterium*, and *Bacteroides* [198]. Probiotics are valuable agents in re-establishing a more favourable microbial balance. 1,4-Dihydroxy-2-naphthoic acid (DHNA), obtained from probiotics, shows anti-inflammatory and antitumorigenic properties by triggering intrinsic apoptotic pathways and downregulating oncogenic proteins. DHNA encourages cell cycle arrest, enables apoptosis, and inhibits metastasis in prostate carcinoma cell lines [197]. In prostatic co-culture designs, *Bifidobacterium longum* and *B. psychaerophilum* decreased oxidative damage and markers of inflammatory disorders while simultaneously normalising androgen levels. This suggests that they have the potential to alleviate benign prostatic hyperplasia (BPH) and potentially prostate cancer [199]. Although there is promising preclinical evidence, there is a lack of clinical data on the use of probiotics to prevent or treat prostate cancer. The majority of positive results have been obtained from preliminary or early-phase studies, and more substantial clinical trials are required [85,198].

### 3.7. Pancreatic Cancer

Probiotics have gained consideration as a potential treatment adjuvant for pancreatic cancer. According to the findings of recent studies, certain strains of probiotics have the potential to make the lives of pancreatic cancer patients more pleasant by mitigating the side effects that are often linked with treatment [200]. When used together with gemcitabine chemotherapy, multistrain probiotics (*Lactobacillus paracasei* and *Lactobacillus reuteri*) decreased tumour growth, reduced signs of cell growth, and improved liver health in mice with pancreatic cancer. Furthermore, probiotics alone revealed the ability to reduce precancerous changes, demonstrating that they possess the potential to prevent both direct and adjuvant antitumour activity [201]. According to a retrospective clinical study, that patients with pancreatic cancer who consumed oral probiotics during palliative chemotherapy had a higher overall survival rate than controls, which may have a beneficial effect on patient outcomes [202]. Probiotics or synbiotics (probiotics plus inulin) given perioperatively to patients with pancreatic cancer surgery improved tumour-infiltrating CD8+ T cells, increased IFN-γ expression, decreased inflammatory cytokines, and lessened postoperative complications like anastomotic leakage and bacteraemia, according to a randomised controlled trial [203].

It has been established that compounds derived from probiotics, such as ferrichrome from *Lactobacillus casei* and heptelidic acid from *Aspergillus oryzae*, have the ability to prevent the growth of pancreatic cancer cells both in vitro and in vivo. The ways these effects occur include turning on p53, interrupting the cell cycle, triggering cell death, and activating the p38 MAPK pathway. Additionally, probiotic strains and their metabolites have the ability to reprogramme tumour-associated macrophages, boost the infiltration of CD8+ T cells, and improve the effectiveness of immunological checkpoint inhibitors. This is accomplished by activating pathways such as TLR4, which ultimately results in enhanced antitumour immunosurveillance [150,204]. Additionally, the research indicates that *Lactobacillus rhamnosus* GG accompanied by gallium-polyphenol network (LGG@Ga-poly), has the potential to improve immunotherapy in the treatment of pancreatic cancer by altering the interactions between the microbiota and the immune system. Due to the fact that it can particularly target pancreatic tumours, LGG@Ga-Poly is able to eliminate tumour-promoting proteobacteria and microbiota-derived lipopolysaccharides, decrease the production of immunosuppressive PD-L1 and interleukin-1β, decrease immunotolerant myeloid populations, and enhance T lymphocyte infiltration [205]. Probiotics have a significant role in decreasing the growth of pancreatic tumours and escalating the efficacy of chemotherapy and immunotherapy, improving patient outcomes.

In general, probiotics have been shown to reduce the adverse effects of chemotherapy and radiation therapy (particularly diarrhoea), improve the microbiota in the gut, reduce the incidence of infections in cancer patients, and improve surgical results [206,207]. *Bifidobacteria* and *Lactobacillus* are two of the most frequently utilised strains, and they are frequently combined in multistrain formulations [47,207]. It has been suggested by studies that the optimal dosages of probiotics are typically between 10^8^ and over 10^10^ CFU per day based on the particular strain and health issue that is being addressed; treatment durations are typically between 6 and 8 weeks or longer, and the most common schedules are either daily or twice daily. However, specific optimal regimens vary depending on the condition, and additional research is required to provide precise recommendations [208,209,210,211]. It is difficult to find evidence regarding the appropriate dosage, duration, and timing. The vast majority of reviews and meta-analyses highlight the importance of conducting more standardised clinical trials on a larger scale to ascertain these criteria.

## 4. Probiotics Synergistic Relation with Chemotherapy

Studies propose that probiotics synergise with chemotherapy or immunotherapy through modulating the gut microbiota to increase the production of beneficial metabolites (such as butyrate), promoting the infiltration and activation of cytotoxic T cells, reducing the function of regulatory T cells, remodelling the microenvironment of the tumour (including shifting macrophage phenotypes and loosening the extracellular matrix), metabolising immunosuppressive molecules (such as adenosine), and locally delivering immunostimulatory agents or checkpoint inhibitors [212,213,214,215]. All of these actions contribute to an improvement in immune responses and the efficacy of the drug. Probiotics exhibits their action through multiple mechanisms to prevent the development and spread of cancer directly and indirectly and synergistically with anticancer drugs. This consists of colonising and defending the gastrointestinal tract (GIT), triggering autophagy and apoptosis, generating metabolites, preventing metastasis, reducing inflammation, boosting the efficiency of immune checkpoint inhibitors, enhancing the infiltration of T cells particular to cancer, and stopping the cell cycle [195]. Numerous anticancer medications, such as cyclophosphamide, cisplatin, trastuzumab, 5-fluorouracil, nivolumab, apatinib, tamoxifen, irinotecan, and gemcitabine, have been shown to interact synergistically with probiotics. Probiotics and anticancer medications together have the potential to control drug resistance, minimise side effects, inhibit recurrence, and reduce treatment expenses [195,201].

Probiotics synergistically enhance the immune response by stimulating Toll-Like Receptors (TLRs) and interacting with NOD-Like Receptors (NLRs). Probiotics stimulate TLRs, enhancing the innate immune response, which is crucial for augmenting antitumour immunity [216,217]. Simultaneously, the activation of NLRs influences inflammatory responses and facilitates several immune functions aimed at tumours. This collaborative activity produces a synergistic impact, enhancing the body’s capacity to resist cancer more effectively than either mechanism could accomplish independently. Consequently, probiotics not only augment immune function but also synergise with these receptors to maximise the comprehensive antitumour response [218,219]. Through decreasing pro-inflammatory cytokines like TNF-alpha and IL-6 and increasing the generation of cytokines that suppress inflammation like IL-10, some probiotic strains develop the immune response. In addition, by activating caspase and releasing cytochrome C, these strains can cause tumour cells to undergo apoptosis by the intrinsic pathway. Enhancing apoptosis and modifying cytokine levels synergistically enhance antitumour efficacy [220,221,222]. Probiotics’ overall therapeutic effectiveness in the treatment of cancer is synergised by this combination.

## 5. Probiotic-Derived Metabolites as Predictive Biomarkers

There is a growing collection of research suggesting that particular metabolites that are produced by probiotics and the larger gut microbiota can function as predictive biomarkers for the response to cancer treatment. The role that SCFAs like butyric acid play in modifying immunological responses is increasingly being recognised. In particular, the significance that SCFAs play in boosting the effectiveness of immunotherapies like checkpoint inhibitors is currently being recognised. Studies have demonstrated that immunotherapy responders and non-responders exhibit varied levels of SCFA expression, which suggests that these SCFAs have the potential to serve as prognostic biomarkers for the effectiveness of treatment [223,224]. Furthermore, it has been demonstrated that the administration of probiotic supplements such as *Lacticaseibacillus rhamnosus* Probio-M9 has the ability to regulate the metabolites in the gut and boost the immune responses against tumours. Particular metabolites, such as butyric acid, α-ketoglutaric acid, N-acetyl-L-glutamic acid, and pyridoxine, have been found to correlate with enhanced treatment outcomes [224]. Metabolites generated from probiotics exhibit potential as noninvasive biomarkers for the early identification of cancer. Engineered probiotics can be designed to generate specific metabolites that can be detected in urine, facilitating the identification of tumour presence, as evidenced in liver metastasis models [225]. In breast cancer, computational analyses indicate that specific probiotic metabolites (e.g., succinate, cadaverine, and p-cresol derivatives) may function as molecular biomarkers for diagnosis [226]. Microbial metabolites, including short-chain fatty acids, bile acids, and indole derivatives, have been associated with colorectal and gastrointestinal malignancies and are under investigation as potential early diagnostic and prognostic indicators [226,227,228]. Through the remodelling of the tumour microenvironment, the regulation of immune cell activity, and the modulation of critical signalling pathways involved in cell proliferation, apoptosis, and inflammation, probiotic metabolites have an impact on the progression of cancer and the disease treatment that is being administered [228,229]. These metabolites can be found in biofluids, for instance, urine, serum, and cystic fluid, which facilitates risk assessment, prognostication, and monitoring of treatment response. This is particularly beneficial in malignancies such as pancreatic ductal adenocarcinoma [230].

Even though the potential of metabolites obtained from probiotics as biomarkers is encouraging, the evidence that is now available is inconsistent, and the majority of the findings are not yet applied for routine clinical application. There is a wide range of diagnostic performance, and more validation in large-scale studies is required [227]. Due to the fact that the effects of microbial metabolites vary depending on the situation, personalised techniques that take into account individual microbiome profiles and cancer types are highly recommended [229,230].

## 6. Clinical Studies

Probiotics have been evaluated as adjunctive therapies in cancer treatment in recent clinical trials, with promising outcomes in a variety of settings. For example, a study conducted by Zheng et al. demonstrated that a particular probiotic compound substantially reduced inflammation and improved immune recovery in patients who were undergoing gastrectomy for gastric cancer. This study suggests that the compound has the potential to be used as an adjuvant treatment in this population [231]. Another fascinating trial examined the impact of *Clostridium butyricum* strain MIYAIRI 588 (CBM588) on patients with metastatic renal cell carcinoma. The results suggested that CBM588 could extend progression-free survival when administered in conjunction with ipilimumab and nivolumab, demonstrating the potential of probiotics to improve the efficiency of immunotherapy [231]. It was discovered through a comprehensive review and meta-analysis of five trials that included a total of 435 patients that probiotics have the potential to be useful in the prevention and treatment of oral mucositis (OM) brought on by cancer treatment. The findings indicate a considerable degree of variability; nonetheless, the utilisation of probiotics resulted in a reduction in the risk of OM for grades three and higher as well as for all grades. In terms of the percentage of patients who completed their cancer treatment, there was no discernible difference between probiotics and placebo [114]. According to the results of a broad analysis of randomised controlled trials, probiotics, particularly strains of *Lactobacillus* and *Bifidobacteria*, improved the patients’ distress due to colorectal cancer. They improved the productivity of the gut microbiota, reduced the complications that were linked with postoperative infections, and prohibited the pro-inflammatory cytokines generation. Moreover, probiotics were reported to decrease the chemotherapy-induced adverse effects, improve surgical effects, and lower the mortality risk [47].

In mono-strain preparations, the focus is on a single probiotic strain that has been evaluated by considerable research to discover the specific health effects this strain possesses. As a consequence of this, the results of these preparations are more anticipated, and a greater understanding of probiotic strain efficacy is achieved. On the other hand, mono-strain products concentrate on a single probiotic strain that has been subjected to extensive research to determine the precise health effects it delivers. Certain strains may improve immunological responses or decrease inflammation, and both types are essential in cancer treatment. On the contrary, multistrain preparations merge numerous probiotic strains, providing potential advantages via synergistic interactions. This approach may recover overall efficacy by addressing various pathways concerned in the development of cancer and response to treatment [232]. Clinical trials have established different outcomes regarding the efficacy of various formulations. Various studies have shown that multistrain probiotics encourage gut health and enhance the efficiency of cancer therapies, including chemotherapy, by improving side effects such as diarrhoea and improving overall quality of life [233]. Probiotics, combinations particularly *Lactobacillus* and *Bifidobacterium*, have been revealed to be effective in controlling dyslipidaemia and obesity in breast cancer patients; as found in a comprehensive review and meta-analysis of clinical trials, they reduce pro-inflammatory markers such as TNF-α, later improving the quality of life in those who are identified with breast cancer-associated lymphedema [189]. The intricacy of multistrain formulations may also result in diversity in safety profiles; subsequently, interactions among different strains could cause unforeseen adverse effects [234]. Probiotics, as dietary supplements, lack strict standards for efficacy and safety certification. While some strains have been experimentally supported, their health-promoting effects have not been proven. Large-scale clinical trials are needed to identify beneficial strains for cancer prevention and treatment. The challenge is to use probiotics and their products to regulate patient flora, potentially using intestinal flora as a cancer biomarker [145].

## 7. Protection from Chemotherapy-Induced Toxicity

Clinical management of various cancers through chemotherapy and radiotherapy is linked to various side effects and toxicity [235]. As a result, there is always an unmet need to investigate agents that reduce these risk factors. Natural products have gained popularity due to their potent antioxidant and antitumour properties. Previously, some groundbreaking findings revealed that numerous bacteria in the human intestinal gut promote growth while preventing pro-carcinogens from turning into carcinogenic substances. As a result, probiotic-integrated approaches are currently being investigated as rationalized therapeutics in cancer treatment [236].

Probiotics taken orally have been used to enhance the gut microbiome, which has been shown to mitigate the negative effects of chemotherapy and its gastrointestinal complications, including mucositis and diarrhoea [237,238,239]. Probiotic strains such as *Lactobacillus casei*, *Lactobacillus delbrueckii*, *L. rhamnosus*, *L. acidophilus*, *Bifidobacterium bifidum*, *Bifidobacterium breve*, *Bifidobacterium infantis*, *Bifidobacterium lactis*, etc., have demonstrated potential in lessening the severity of mucositis caused by chemotherapy [240]. *L. acidophilus* probiotic strains have been shown to be useful in treating different aspects of toxicity brought on by chemotherapy. In the setting of chemotherapy, *L. acidophilus* has shown anti-inflammatory properties and enhanced intestinal barrier function [241,242]. It has been demonstrated that *L. rhamnosus* lowers the frequency and intensity of diarrhoea brought on by chemotherapy [243]. Multiple species of the *Bifidobacterium* genus have been reported to reduce nausea and vomiting brought on by chemotherapy [244]. Probiotics could be helpful in protecting the liver by lowering inflammation along with oxidative stress, as chemotherapy sometimes may lead to liver toxicity. In animal studies, it was discovered that some probiotic strains, such as *L. rhamnosus*, reduce liver damage brought on by chemotherapy [245].

A clinical study conducted in 2014 on patients with pelvic malignancy found that probiotics (*L. acidophilus* and *Bifidobacterium longum*) prevented moderate or severe diarrhoea caused by radiation therapy in 35% of probiotic-using patients in contrast to 17% in the group receiving a placebo [246]. Additionally, a clinical study conducted in 2015 examined the effectiveness and safety of a probiotic formulation containing several strains of bacteria, such as *Lactobacilli* and *Bifidobacteria*, among patients undergoing chemotherapy based on irinotecan for colorectal cancers. When compared to the placebo group (60.9%), the study revealed a decline in the overall rates of diarrhoea among patients receiving probiotics (39.1%). In a randomized clinical trial of *Saccharomyces boulardii* in patients with colorectal cancer going through colon resection, there was a significant decrease in pro-inflammatory as well as anti-inflammatory cytokines in the intestinal mucosa [247] in contrast to 8.7% in the group given placebo in a study carried out with patients receiving chemotherapy [246]. These multistrain probiotics may offer a broader strategy to improving the gut microbiome and managing chemotherapy-related side effects. It is essential to note that the particular kind of probiotic and dosage may differ based on the patient, chemotherapy type, and extent of the side effects. Furthermore, probiotics may be more or less effective during chemotherapy depending on when and how long they are taken. Probiotics are generally advised to be taken continuously during the course of treatment and the recovery phase, beginning prior to the start of chemotherapy. Overall, using particular probiotic strains and formulations can be a helpful adjuvant therapy in treating the different aspects of toxicity brought from chemotherapy.

## 8. Safety Considerations of Probiotics in Cancer Therapy

Concentrating on safety becomes essential when considering about using probiotics to treat cancer. Immune systems in cancer patients are frequently weakened by the disease itself or by side effects of radiation and chemotherapy [20,248]. While some probiotic strains might be safe, others might raise the risk of bacteraemia or sepsis, especially in those who are already vulnerable. An accurate assessment of probiotic treatment and/or consumption is crucial for managing illness [249]. The subject of sustained probiotic utilisation in neutropenic cancer patients has been the subject of therapeutic interest due to concerns regarding the risk of infection in immunocompromised individuals. This demographic has not shown any remarkable adverse effects, which reveals that specific probiotic strains may be safe and well tolerated by this population. The evidence that is presently available comes from tiny pilot studies. During the course of a pilot trial that utilised *Enterococcus faecium* M-74 in patients suffering from solid and haematological malignancies, including those with severe neutropenia, the probiotic strain was not responsible for any infections or febrile episodes that might have occurred. The treatment was well tolerated, and there were no remarkable adverse effects observed [250]. In randomised controlled trials, there were no significant adverse effects or serious infections (including bacteraemia) recorded between the probiotic and placebo groups in cancer patients, even those with neutropenia [238,251,252]. There are no studies that revealed major adverse events or deaths linked to the use of probiotics, according to a large systematic review comprising 12 trials with a total of 1554 participants. Myocardial infarction was the only cause of death that was reported, and it was unrelated to the application of probiotics [252]. Small clinical trials conducted on cancer patients of both paediatric and adult ages who were taking chemotherapy showed that the administration of probiotics was not linked to increasing the incidence of infectious problems or severe side effects [238,251]. When used as a therapeutic agent, evaluating the safety aspects of probiotic consumption is a difficult yet essential task. The stability, dosage, and purity of the probiotic products are important factors to consider in both clinical and experimental research on the therapeutic efficacy of probiotics [253,254]. It is important to choose the right probiotic strains because not all of them have had enough research performed on their safety in cancer patients. As a result, it is critical to use strains supported by clinical studies illustrating their safety profile in this particular population. Probiotics and cancer therapies may also interact, impacting the effectiveness of treatment or producing unfavourable side effects. For example, probiotic-induced modifications to the gut microbiota may affect how well some oral medications are absorbed [85,238].

**Figure 10:**
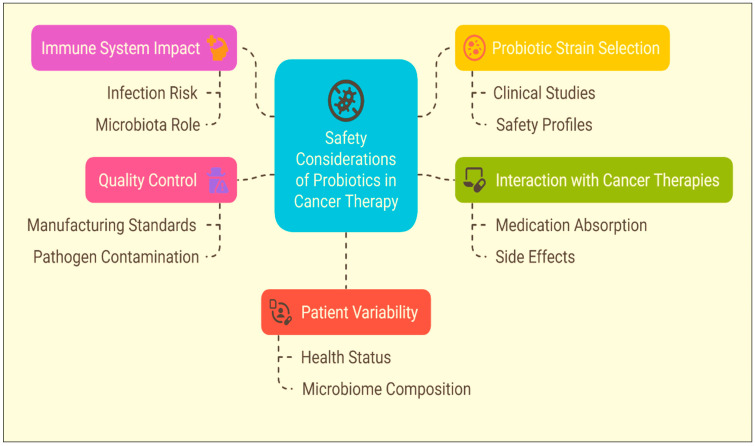
Exploring the Safety of Probiotics in Cancer Care: Important factors to take into account include the health of the immune system, particular strains, interactions between treatments, patient variability, and quality control to manage pathogen contamination during formulation.

One of the most frequent toxicities linked to cancer that causes the chemotherapy regimen to be discontinued or lowered is diarrhoea brought on by the treatment [248]. Cancer patients who experience diarrhoea may find it uncomfortable and experience a decreased response to radiation and chemotherapy. They may also need additional care to avoid related morbidity and mortality [255]. Since infections are common in patients with compromised immune systems, the microbiota plays an important role in immunity [248,256]. Probiotics should be assessed for safety as well as efficacy in combating infection. It is especially important to look into the possibility that probiotics could become infected themselves. Quality control is another major concern, as variations in manufacturing standards can result in inconsistent product quality. Poorly manufactured probiotics may contain harmful pathogens. Common side effects include gastrointestinal issues such as gas, bloating, and diarrhoea, which can be difficult for patients who already have digestive sensitivities as a result of their treatments. Finally, patient variability means that factors such as health status, microbiome composition, and treatment plans can influence how safe or well-tolerated the probiotics are. This demonstrates the significance of obtaining tailored guidance from healthcare specialists when integrating probiotics into cancer treatment regimens. Ongoing study is necessary to determine the comprehensive safety and efficacy of probiotics in these situations (Figure 10).

Considering safety, several techniques have been proposed to potentially ensure the viability of probiotic colonization at target sites. One of the major challenges is to ensure the survival of probiotics in the unfavourable condition of the gastrointestinal tract and that they effectively colonise target regions like the colon [257,258]. Encapsulated microparticles and enteric-coated capsules are both sophisticated delivery approaches proposed to preserve probiotics and develop their targeted release. When compared to conventional enteric-coated capsules, encapsulated microparticles—mainly using prebiotics and sophisticated polymers—show compelling evidence of improved protection and targeted colonisation. Microparticles made from materials like polymethacrylates, alginate-chitosan, casein-chitosan, and starch-based nanoparticles effectively shield probiotics from stomach acid and bile, ensuring they stay alive as they pass through the stomach and small intestine [258,259,260]. Enteric-coated capsules are made to tolerate stomach acid and dissolve in the higher pH of the gut, thereby preserving the probiotics until they reach t the colon [261]. Novel coatings (e.g., Eudragit S and Acryl-EZE) deliver prolonged release; nonetheless, their protective capabilities are typically less configurable than those of microparticle systems. These capsules are valuable and can be made in large numbers; however, they might not offer the precise, long-lasting release or the ability to stay in the colon compared to advanced microparticle technologies [258].

## 9. Conclusions

Throughout the entirety of this study, the expanding array of scientific information that demonstrates the use of probiotics in cancer treatment and prevention has been taken into consideration. The mechanisms of action that have been proposed include the modification of the gut microbiome, suppression of carcinogen pathways, promotion of immune response, and protection against treatment-related side effects. Studies conducted in both preclinical and clinical settings have demonstrated that a wide range of probiotic strains, including species of *Lactobacillus* and *Bifidobacterium*, have anticancer properties through a variety of different mechanisms. Among these are the induction of apoptosis in cancer cells, the inhibition tumour growth, and the mitigation of adverse effects of radiation and chemotherapy. Clinical results can be influenced by variables like probiotic strain, dosage, time of administration, and unique patient characteristics. Furthermore, more investigation is needed to pinpoint the precise mechanisms through which probiotics affect the immune system and gut microbiota in relation to cancer. The progress of novel technology and different formulation are projected to facilitate a deep link between probiotics, the host, and the gut. A very large human database is required to search for various strains for the prevention and treatment of different types of cancer. Probiotics are generally well tolerated and have a minimal chance of side effects in terms of safety. There have been infrequent reports of probiotic-associated infections in immunocompromised patients and those with serious underlying medical conditions, so caution is advised in these groups. In conclusion, recent research suggests that probiotics could be helpful as a supplemental approach to managing and preventing cancer. Overall, a multifaceted probiotics approach may improve gut health, reduce treatment-related side effects, and boost antitumour immunity. To determine the best probiotic therapies and include them in comprehensive cancer therapy, more study will be needed.

## Data Availability

No new data were created or analysed in this study.
